# Talk the Walk: Does Socio-Cognitive Resource Reallocation Facilitate the Development of Walking?

**DOI:** 10.1371/journal.pone.0156351

**Published:** 2016-06-01

**Authors:** Ronny Geva, Edna Orr

**Affiliations:** 1 Department of Psychology, Gonda Multidisciplinary Brain Research Center, Bar Ilan University, Ramat Gan, Israel, 5290002; 2 The Developmental Neuropsychology Lab, Bar Ilan University, Ramat Gan, Israel, 5290002; University of Portsmouth, UNITED KINGDOM

## Abstract

Walking is of interest to psychology, robotics, zoology, neuroscience and medicine. Human’s ability to walk on two feet is considered to be one of the defining characteristics of hominoid evolution. Evolutionary science propses that it emerged in response to limited environmental resources; yet the processes supporting its emergence are not fully understood. Developmental psychology research suggests that walking elicits cognitive advancements. We postulate that the relationship between cognitive development and walking is a bi-directional one; and further suggest that the initiation of novel capacities, such as walking, is related to internal socio-cognitive resource reallocation. We shed light on these notions by exploring infants’ cognitive and socio-communicative outputs prospectively from 6–18 months of age. Structured bi/tri weekly evaluations of symbolic and verbal development were employed in an urban cohort (N = 9) for 12 months, during the transition from crawling to walking. Results show links between preemptive cognitive changes in socio-communicative output, symbolic-cognitive tool-use processes, and the age of emergence of walking. Plots of use rates of lower symbolic play levels before and after emergence of new skills illustrate reductions in use of previously attained key behaviors prior to emergence of higher symbolic play, language and walking. Further, individual differences in age of walking initiation were strongly related to the degree of reductions in complexity of object-use (r = .832, p < .005), along with *increases*, counter to the general reduction trend, in skills that serve recruitment of external resources [socio-communication bids before speech (r = -.696, p < .01), and speech bids before walking; *r* = .729, *p* < .01)]. Integration of these proactive changes using a computational approach yielded an even stronger link, underscoring internal resource reallocation as a facilitator of walking initiation (r = .901, p<0.001). These preliminary data suggest that representational capacities, symbolic object use, language and social developments, form an integrated adaptable composite, which possibly enables proactive internal resource reallocation, designed to support the emergence of new developmental milestones, such as walking.

## Introduction

Bipedalism, the ability to walk upright on two-feet, has been considered to be a trait defining hominin lineage. It is present in the behavioral repertoire of multiple organisms, often as a volitional goal-directed behavior and not as a default mode of locomoting [1]. It has been suggested that human walking on two feet is ‘an exploitation of a locomotor behavior retained from the common great ape ancestor’[2]; yet, the mechanisms that enabled the transition to habitual terrestrial bipedalism are unknown. Recent dynamic system’s theory [3], synthetic robotics approaches [4], and evolutionary biology [5, 6] suggest that interactions between and within modalities increase the likelihood of the emergence of a new motor achievement. These interactions may encompass cognitive adaptations as facilitators of the emergence of walking on two feet in young infants. To date, no integrative developmental model for the affordance of walking initiation has been proposed and little is known on the potential relations between mental resources and walking.

Research in other species thus far has focused on dramatic anthropometric changes and increased strength as stimulators of gross motor development and initiation of novel modes of locomotion [7], and explored less the role of proactive cognitive development in driving such changes. At the same time, developmental research has mostly explored cognitive developments that are enabled by altered motor experience, and has under-studied potential socio-cognitive changes that may be linked proactively to walking emergence.

We were interested in exploring a potential internal processor that may facilitate the emergence of a new behavior, such as walking. We posulate that "an adaptable composite" programmed for self-adaptation may be an important component in enabling the opportunity for the evolution of a new behavior. This metaphoric term, borrowed from software engineering, describes systems that are able to modify their own behavior and/or self-restructure in response to their perception of the environment, the system's charateristics, and its goals [8], in order to enable self-adaptation in dynamic conditions [8] and prevent network over-load [9].

Given that at any time point in development infant's resources are finite and limited, we postulate that new developments are facilitated by such an adaptive composite, programmed to drive proactive changes in previously attained skills in anticipation and prepration for new developments. More specifically, we suggest that an adaptable internal representational composite possibly supports the emergence of new milestones, such as walking, *proactively*- through resource reallocation.

We sought to gain insight into this framework by exploring infants' mental resource development during the year at which they make the transition from crawling to walking; postulating that the relationship between cognitive development and walking is a bi-directional one. Thus, we explored the under-studied direction of this relationship, that the frequency of language, symbolic play and social bids prior to the emergence of walking would be related to the age of walking initiation. The main notion explored in the current study was that internal resources and the ability to reallocate mental resources would be linked to age of walking emergence.

The developmental psychology literature thus far has not yet identified which mental resources are adaptablly reallocated to support walking initiation. Evolutionary and zoological research point to specific representational changes, namely involving tool use and socio communicative behaviors that may be relevant to walking emergence. Ontogenetic inductions from evolutionary work are not necessarily straight forward. Still, evolutionary research points to behavioral changes in tool use as playing a role in adapting towards habitual bipiedalism. Advancements in evolutionary research suggest that the humanoid shift to an erect posture, which allowed for free hands and developments in tool-use [10], offered necessary advantages in conditions with limited environmental resources [1]. The later tool-use development has been considered by some to be important in enabling mankind the transition to habitual, terrestrial bipedalism [11]. Drawing on ontological-evolutionary similarities, it may be relevant to consider that the proposed adaptive internal resource reallocation composite may incorporate changes in symbolic object use in affording the emergence of infant walking.

The other postulated component in this representational self-adapting composite is changes in socio-communication behaviors. Zoological research points to socio-communicative adaptations in relation to volitional bipedalism, and though cross-species comparisons are tentative, they possibly point to a potential direction. Directions were such that higher propensities for volitional shifts from quadrupedality to facultative bipedality are seen in social-communicative species, such as primates [12, 13], trained Japanese macaques [14] and Capuchin monkeys [15]. Some findings suggest that bipedality has advantages in establishing social standing, and that social considerations are important in the establishment of habitual bipedality [16].

Little is known about the proactive changes in representational abilities in relation to the emergence of walking in developing infants. While discrete changes in locomotion, such as the transition to walking on two feet, are easily visible, this is not the case for more covert transitions, such as changes in symbolic abilities. This may explain the almost exclusive focus in the available body of research on one direction of the trajectory, that walking elicits cognitive development; rather than exploring the reciprocal notion, that alterations in socio-cognitive output may play a role in the initiation of walking. Developmental research has shown that acquiring a new gross motor skill is accompanied by real time changes in rates of expressed behaviors in other domains of functioning [7, 17, 18]. Examples include relations between sitting and object exploration [3, 19, 20], and novice crawling and restricted spatial field scanning [21]. However little is known about *changes* in cognitive behaviors that *precede* and support the emergence of a new motor skill. A novice walker, for example, is required to plan their future movements [1, 22, 23], and thus their strategy choices reflect a need to invest cognitive resources (and the difficulty in doing so) [24, 25]. Adolph and collegues [26, 27] indicated that novice walking infants are not necessarily considering the relations between their own bodies, their skills and the relevant properties of the environment in deciding if an action such as a descent is possible or impossible [24], managing at first less challenging terraine better than more complex ones, with the latter resulting in frequent stumbing and falls [28]. These data possibly point to the notion that mental representation change is not *necessary* for the emergence of infant walking, but is possibly supporting its emergence and its stabilization. That is, novice walkers manage better with less falling in familiar simple terraines, which require less representational resources; and fail in trials that require integrating walking and higher-order symbolic representations, such as in cases with gaps, bridges, etc. [28]. This may support the notion that some adaptations in use of symbolic behaviors are first important, proactively, to free much needed mental resources and enable a relatively smooth transition to walking (as the transition to walking itself is postulated to take up resources); and thereafter enable to manage conditions that probe more representational resources (when walking no longer requires as many resources). This suggests a possible rebound in the degree of use of previously attained representational skills in experienced walkers.

Developmental research exploring samples drawn from the general population has thus far noted specific cognitive changes that occur when walking emerges [29, 30]. We were particularly interested in object use and socio-comunicative behaviors as possible relavent resources supporting walking initiation, as suggested by evolutionary and zoological research. Speech and social bid increases were seen in infants during transitions from stance-and-swing phases [5] to smooth walking [7, 31]. This possibly suggests that some cognitive reductions precede the emergence of walking, freeing available resources in preparation for management of effortful trials of a novel milestone, followed by a "recovery" period in which the resource returns.

As for object use, one important cognitive function that develops at around 6–18 months is object-use in symbolic-play, i.e., use of an object or activity with objects to represent something else. Expressions of symbolic abilities build upon representational resources that evolve gradually from single-object play and single-object sequences to multi-object play and multi-object sequences [32]. These mental representations require especially taxing planning resources, as they entail holding multiple items and maintaining multiple action sequences in the working memory system [33]. Thus, the development of symbolic abilities and socio-comunication in human infants possibly *precedes and supports* walking initiation through resource reallocation.

Examples of real time decreases in certain cognitive skills were previously reported during a transition from one motor milestone to another. Adolph and Tamis‐LeMonda [29] noted a decrease in looking at distant objects in favor of proximal exploration in real-time during the transition from sitting to crawling; but the idea that proactive internal cognitive resource reallocation is linked to the emergence of walking has not yet been explored directly.

Overall, we postulated that internal resource reallocation, expressed by *changes* in frequency of use rather than absolute use levels, would be linked to age of emergence of a novel behavior, such as walking. Specifically, we expected that the development of early and more mature symbolic abilities, which were recently shown to support language development [32], would *precede* walking initiation; yet, there would be a *general tendency to reduce frequency of use* of symbolic outputs in play, verbal communication and social interactions in order to free resources; and finally, that individual differences in *resource reallocation* would be linked to age of initiation of walking.

In considering the research design, models of cognitive knowledge transfer [34] suggest that examination of the resource reallocation notion is possible by using a prospective, longitudinal, multi-faceted design. The current study is a part of a larger research exploring early development from 6–18 months of age [32]. A previous paper focused on symbolic development and language. The current work explored the emergence of walking in the same cohort of infants who were enrolled in a multi-measure prospective study in which they were scheduled to be tested 18 times (*Δt* = bi weekly at ages 6-12m, tri- weekly at ages 12-18m). Through use of this paradigm, we launched a study aiming to explore the following operational hypotheses:

We first postulated that age of initiation of higher-order sequential symbolic play and age of initiation of use of single-word utterances would emerge prior to age of initiation of walking. The second hypothesis postulated that inter-domain reductions in frequency use would be seen prior to emergence of milestone behaviors, such as multi-object sequence symbolic play, initiation of single word expressions and initiation of walking. Both hypotheses were explored using the following variables: frequency of use of verbal expressions [35] (2 levels: babbling and single-word utterances); frequency of social gestures; and frequency of use of simple: [4 levels: simple-order (Single object play, Single object sequences), and higher-order symbolic play levels (Multi-object play and Multi-object sequences)].

The third hypothesis postulated that overall speech and social gesturing would precede, and that their use rate would generally decrease before walking, but children who reallocate resources to speech and social gesturing, contrary to the general trend (having realized the benefit of recruiting external support in preparation of walking), would walk earlier.

The third hypothesis was explored using a *computational approach* postulating zero-sum game notions with regard to resource reallocation (RR), whereby the degree of "free" resources, which serve to recruit external resources would be linked to age of walking initiation. Specifically, the general notion for RR was that it is indeed computable; and that in the case of walking, initiation it would be related to net result of integrating the degree of proactive *decreases* in simple symbolic behaviors at the emergence of higher symbolic play levels and at the emergence of speech; and the degree of *increases* in speech and social bids at the emergence of walking. RR was thus formulated by computing the difference between these key change coefficients:

$$\Delta \;{Sp}_{2}/\Delta t=\left[{Sp}_{2}(T_{w})-{Sp}_{2}(T_{w}-\Delta t)\right]/\Delta t$$

$$\Delta \;S_{ymb2}/\Delta t=\left[S_{ymb2}(T_{l})-S_{ymb2}(T_{l}-\Delta t)\right]/\Delta t$$

$$\Delta \;S_{ymb3}/\Delta t=\left[S_{ymb3}(T_{s})-S_{ymb3}(T_{s}-\Delta t)\right]/\Delta t$$

$$RR=\Delta {Sp}_{2}/\Delta t-(\Delta S_{ymb2}/\Delta t-\Delta S_{ymb3}/\Delta t)$$

**Legend.** *T*_*w*_ *= T at transition to walking; T*_*l*_ *= T at transition to single word utterances; T*_*s*_ *= T at transition to sequential multi-object symbolic play; Δt = Time difference between observations*, *2 week intervals; Sp*_*2*_ *= Rate of speech at level 2 (single word utterances); S*_*ymb2*_ *= Rate of symbolic play at level 2 (single toy sequences); S*_*ymb3*_ *= Rate of symbolic play at level 3 (multi-toy play)*.

## Materials and Methods

### Participants

The study procedure and informed consent process was approved by the ethics committee of Bar Ilan University. Mother-infant dyads were recruited to participate in a prospective multi-measure study. Written consent was signed by all parents. Nine infants (four girls and five boys) were studied bi-weekly for a year, from 6 to 18 months of age. Participants were of middle socio-economic backgrounds living in an urban center of Israel. From 6 to 12 months of age, all ts were presented with a structured paradigm which was videotaped for one hour in their homes every two weeks; and from 12 to 18 months of age, participants were videotaped once every three weeks, resulting in a total of 280 testing sessions. All participants were videotaped with their mothers sitting nearby. Sessions were always coordinated with the mothers and scheduled for mornings or afternoons according to the babies’ states of wakefulness, and always after they had slept and were fed.

### Procedure and Materials

This study is part of a larger study with the same participants on early development from 6–18 months of age [32]. Fifty objects, differing in size, color, texture, shape and function, were presented by the experimenter to the infants. Items included common household infant objects (e.g., a pacifier, bottle, teaspoon, and bowl); some smaller-sized replicas of objects; along with ambiguous items (e.g., a plastic hoop) and 4 different dolls. Evaluation of symbolic resources [35] was administered in 2 development-dependent versions: A) Pre-independent sitting, during which infants were laid supine on a mat found in their home and objects were placed by the experimenter one at a time in the palms of their hands. Objects, that were more appropriate for holding, controlling and manipulating (e.g., doll, bowl, pieces of soft linen, etc.) were suggested to the infant at this stage. Infants who laid supine, most of the time, focused on exploring and playing with the objects and marley bid to their mother that sat nearby; and B) Post-independent sitting, during which the child began in a sitting position and the experimenter asked crawlers and walkers to sit prior to displaying the objects. Once the infant sat all objects were presented and were within reach of the child during each experimental session. Crawlers and walkers were free to move and were not instructed to remain in a sitting position. Mothers and the experimenter sat nearby, approximately 1–2 meters from the infant, enabling the infant to focus on the interaction opportunities. Mothers were instructed to sit nearby and avoid demonstrating item use. Each participant was scheduled to be tested 18 times.

All observed actions were recorded and analyzed. Mother-infant exchanges were usually evoked spontaneously in response to a symbolic act. Vignettes in which an infant directed their gaze straight at the mother or directed an object toward her were coded as an infant requesting a maternal response. Coded maternal responses included: eye-contact, vocalizations from a distance, smiling at the infant, complying with the infant's request [e.g., pretending to eat, using exaggerated gestures and vocalization (e.g., ‘yummy’)], and mimicking or praising the infant (e.g. ‘you are a good cook’)].

Actions were marked as symbolic acts if they included novelty in object use and intentionality [36], and were coded into one of four symbolic level categories using established criteria [35]. The four types of symbolic acts were defined according to the number of objects and actions that could be combined. Symbolic acts were defined as follows: Single-object play (baby holds a single object and performs a single pretend action, such as putting a stick next to the ear for a telephone). Single-object sequences (baby holds a single object and performs several pretend actions, such as holding a placemat over the face to hide and then exposing the face and smiling at the observer) Multi-object play (infant uses several objects and performs single pretend actions, such as placing a bowl on the head of a doll as a hat). Multi-object sequences (infant uses several objects to perform several pretend actions, such as placing several objects into a pot, stirring and then close the pot with a cover).

Classification of symbolic action to four levels was examined for inter-rater reliability by two trained coders. Approximately 50 scenes were examined, 12 for each type of symbolic act, making up about 20% of the total testing sessions. Nominal agreement between the two independent coders resulted in Cohen’s kappa reliability for single object play and multi-object play = 0.90. The reliability for single-object sequence and multi-object sequence was 0.85.

Age of initiation of motor, social, lingual and cognitive behaviors was marked at each testing session in which the behavior was observed for the first time, as follows:

Symbolic level (Single-object play, single-object sequences, multi-object play, and multi-object sequence).

Verbal ability: babbling (produce syllables in reduplicated series of the same consonant and vowel combination, such as: dada,mama); 1-word utterances (evoking single word that directly express either entity (doll) or dynamic meaning (eating) [37, 38]. Babbling and saying one word in target language has different sound patterns, therefore we use infants’ own vocalization repertoire as a landmark for pointing on babbling and speech onset.

Social bid frequency and type. Infant’s spontaneous locomotor status was marked at each visit, irrespective of play, as follows: pre-sitting (lying supine), sitting (infant sits and reaches independently without any support), crawling (moving on hands and knees while belly raised, keeping body balance during alternating movements of arm and legs), and walking (gained independent balanced walking steps adult like while pushing upward with the stationary foot supporting).

Relative changes in frequency of use were then computed to determine resource reallocation. Estimates of resource reallocation were based on ages of initiation of each symbolic, verbal and motor behavior, as well as relative rates of use of each behavior at each time point. We then computed changes in rates of behaviors from one point in development to the next in the 4 interacting domains: locomotion (2 levels), verbal communication (2 levels), use of social gestures (infant initiation and maternal responsiveness) and symbolic play (4 levels) [35], as well as recorded ages of peak frequencies of target behaviors for each participant and recovery of frequency rates past target behavior initiation.

## Results

**Fig 1:**
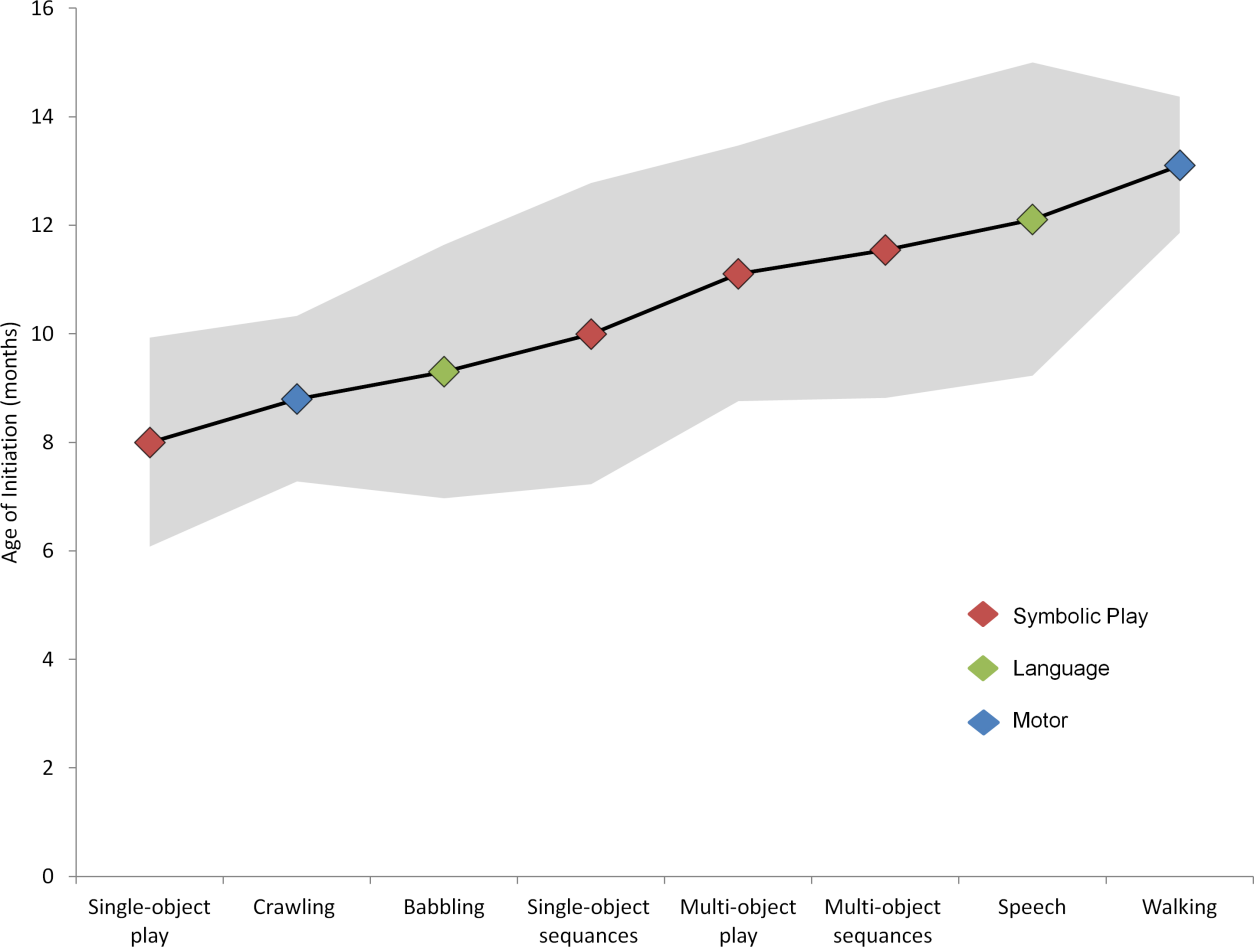
Multi-dimensional developmental sequences of the symbolic level (verbal and gestural) and motor behaviors from 6 to 18 months of age. Mean ages of initiation- black trajectory, SD- in grey shade).

Results of between-subject longitudinal data indicate heterogeneity, with higher-order symbolic-play and early language frequently emerging prior to the onset of walking, in the direction postulated. In order to examine the first hypothesis that attaining all 4 levels of symbolic play and use of single word utterances would precede age of walking initiation, we explored the developmental trajectories of each participant. The trajectories were such that most infants attained *all four* levels of symbolic-play on average 6 weeks prior to walking initiation, namely, in 7 of 9 of the infants who participated in the study). Also, age of initiation of single-word utterances preceded by 4 weeks on average prior to walking in these cases, thereby supporting the first hypothesis (Fig 1).

Mean age of emergence of symbolic play levels, speech milestones and motor milestones (Fig 1) indicate that symbolic abilities verbal and gestural tend to emerge prior to age of emergence of walking.

The second hypothesis pertained to a notion that use of recently attained effortful skills would be reduced prior to attainment of new milestones, indicating a tendency to free resources proactively in order to enable resource reallocation.

**Fig 2:**
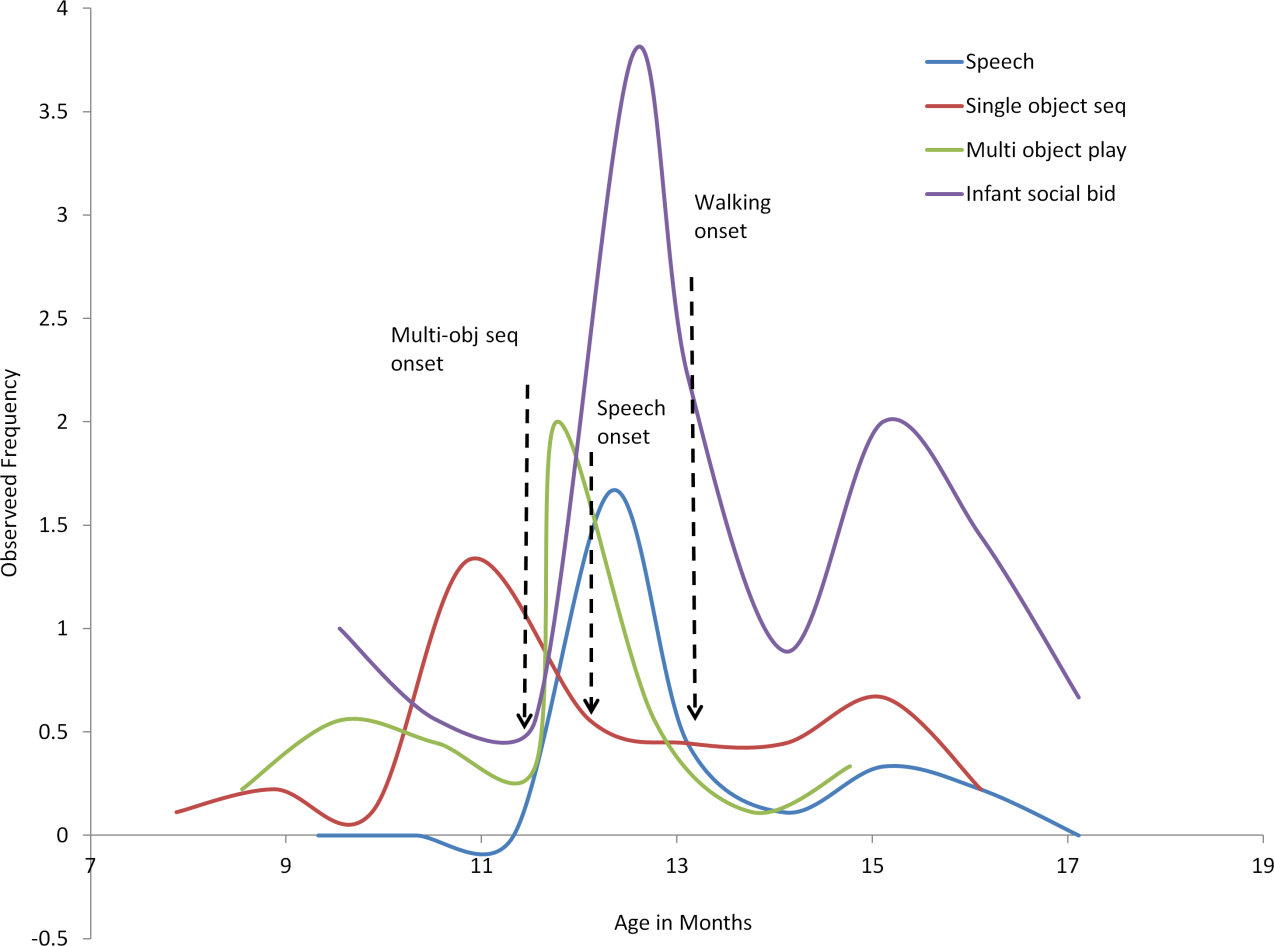
Inter-domain resource allocations. Frequency of use of representational behaviors [at Y axis, symbolic play (in blue and green), verbal communication (in red) and social bids (in purple) before and after age of attainment of key developmental milestones (in symbolic ability, communication and walking, marked by dotted arrows on X axis of age in months).

Mean trajectories were plotted for the participants’ observed frequencies of these behaviors at key milestones (age of emergence of complex symbolic play, age of emergence of speech and age of emergence of walking), as compared with frequencies at preceding, post emergence and peak time points, as a function of age of behavior emergence (Fig 2).

The figure shows that the frequency of use of all coded behaviors is reduced prior to the initiation of walking. Similar, often less dramatic trends are evident prior to the emergence of preceding milestones, with moderated reductions in multi-object play at multi-object sequencing play, significant reductions in simple symbolic play at the emerge of single word utterances, and marked reductions in speech and social bids prior to the emergence of walking,

Specific changes in regard to peak frequencies include: Single object sequence play reduced prior to emergence of language from an average peak of 2 (±.1.6) to an average of .56 (±.0.7) at the emergence of single word utterances, t = 2.393, p<0.004; language use reduced prior to emergence of walking from peak levels of 1.67(±0.7) to 0.44 (±0.73), t = 3.773, p<0.005; and infants social bids before walking reduced by half upon walking emergence, from a peak average of 3.7 (±2.4) to 2.222 (±3.1), t = 3.50, p<0.008).

Evidence for rebounds are also seen after walking initiation (Fig 2), mostly in social bids. Thereby further supporting the notion of a proactive resource reallocation of socio-communicative prior to emergence of walking.

**Fig 3:**
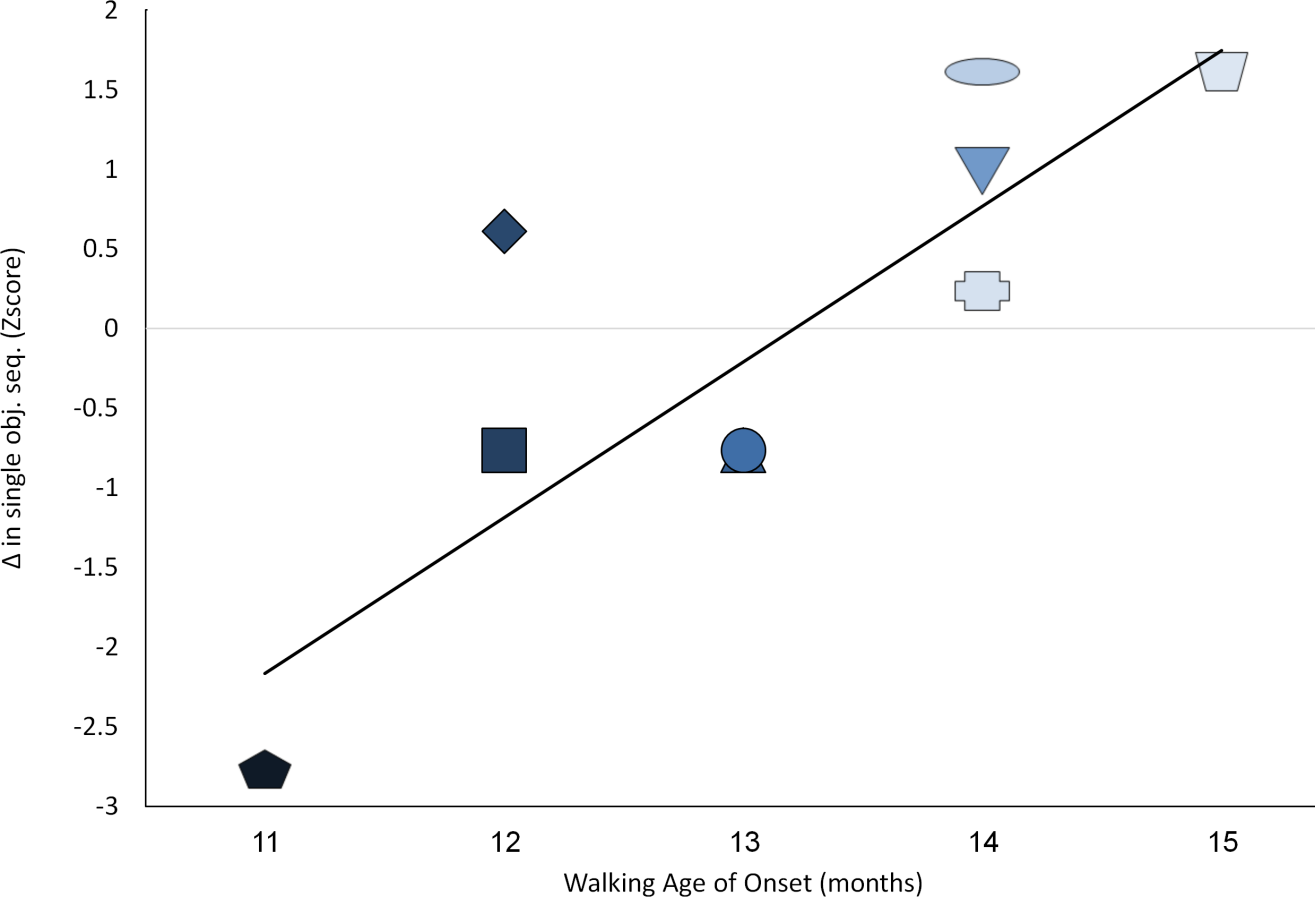
Relations between age of walking initiation and changes in simple object use at that age of speech initiation. Darker color shade for participants represents earlier age of walking initiation; Y-axis depicts standard Z scores.

**Fig 4:**
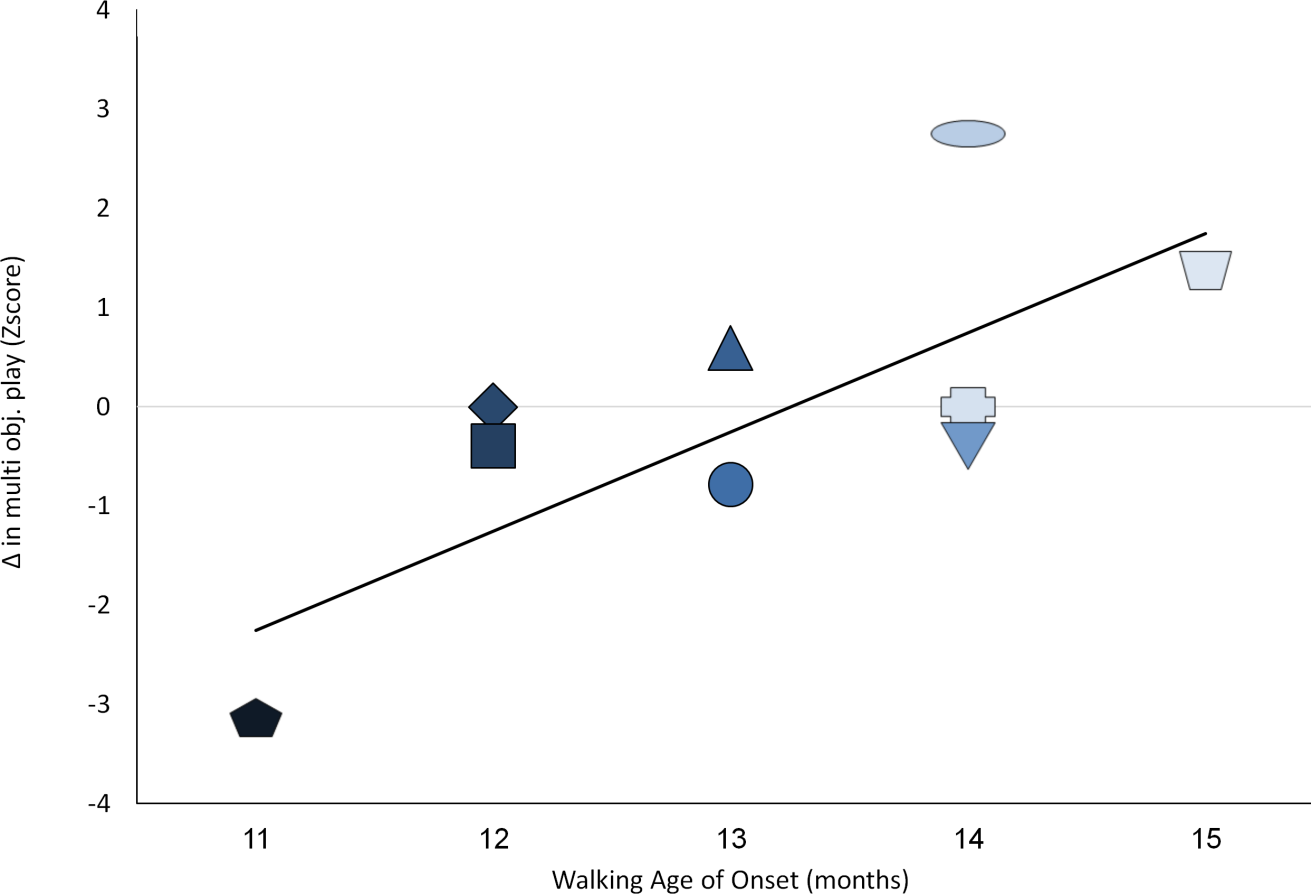
Relations between age of walking initiation and changes in multi-object play before multi-object sequence play emerges. Darker color shade for participants represents earlier age of walking initiation; Y-axis depicts standard Z scores.

We then explored individual differences with regard to the degree of change in resource reallocation and age of initiation of walking. Pearson correlation analysis provided support for the postulated relations. Decreases in simple symbolic sequential play at the transition to *language* (Δ Symb2/Δt) and intra-domain decreases in complex multi-toy play at multi symbolic sequential play (Δ Symb3/Δt), were correlated strongly with age of emergence of walking (r = .832**, p < .005, Fig 3; and r = .730*, p < .01, respectively; Fig 4).

**Fig 5:**
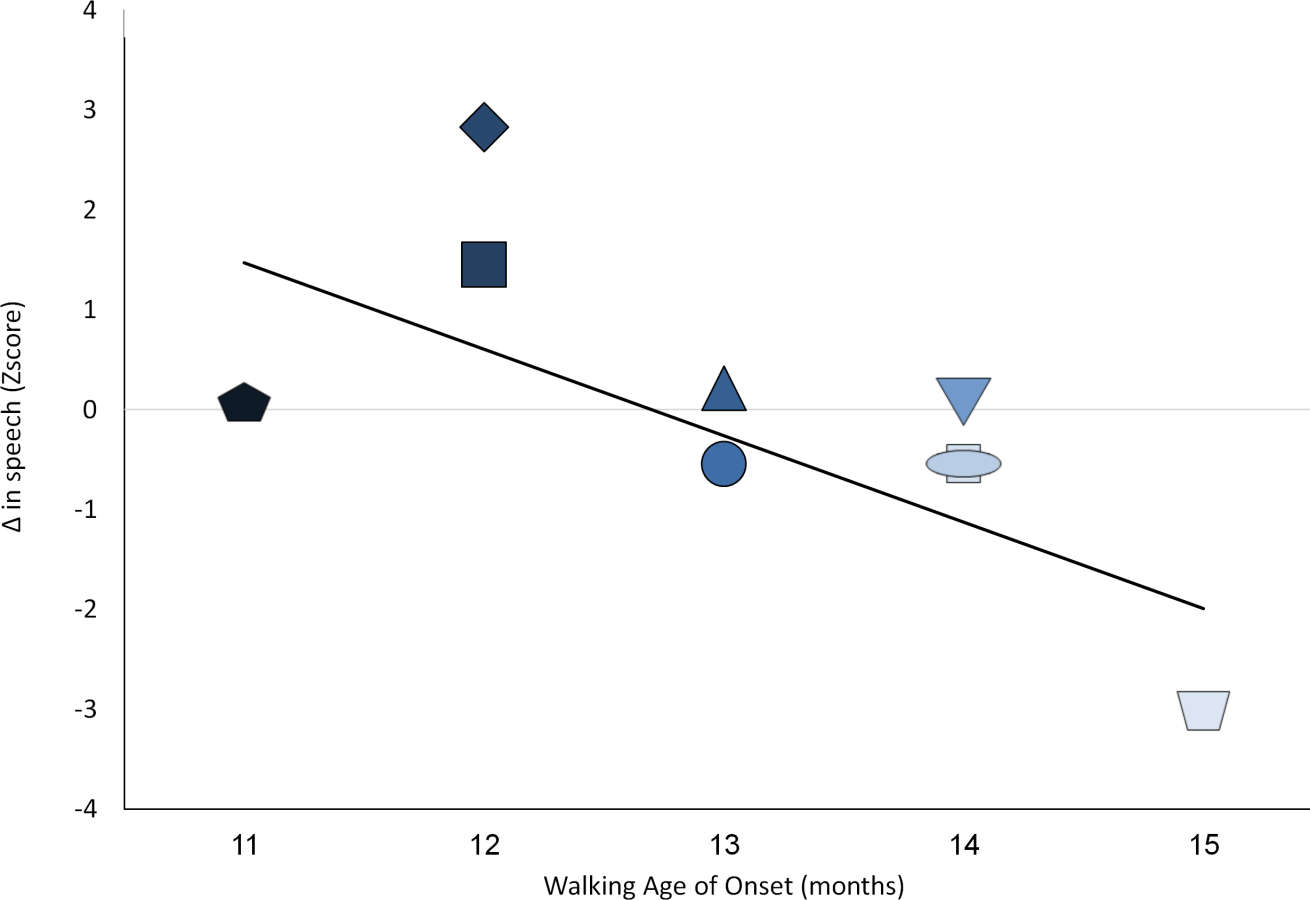
Relations between age of walking initiation and changes in frequency of language at that age. Darker color shade for participants represents earlier age of walking initiation; Y-axis depicts standard Z scores.

These positive relations were complemented by expected opposite relations; such that *increases* in use of speech and social bids, were both linked to earlier onset of crawling and earlier onset of walking (*r* = -.683*, *p*<. 01, and *r* = -.729*, *p* < .01, respectively, Fig 5).

**Fig 6:**
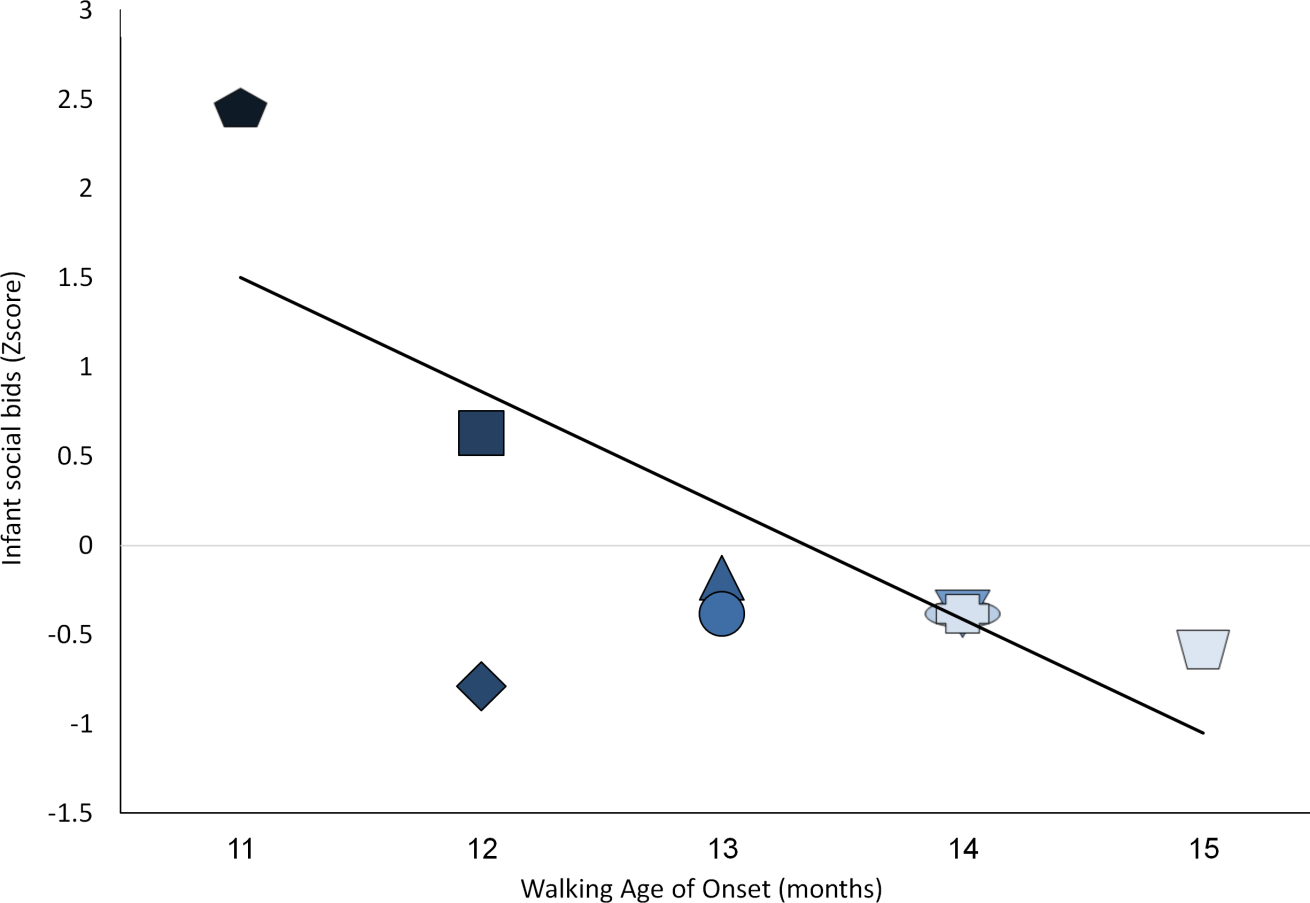
Relations between age of walking initiation and changes in infant and mother social bids at the age of emergence of language. Darker color shade for participants represents earlier age of walking initiation; Y-axis depicts standard Z scores.

Similarly, in exploring social bids, relations between the rate of mother-infant exchanges and the onset of walking also yielded negative correlations in the anticipated direction. Increases in infant requests and corresponding trends in maternal responsiveness prior to speech onset were both strongly associated with earlier walking acquisition (r = -.696*, p < .01, and r = -.712*, p < .01, respectively; Fig 6).

Integrating these relations enabled exploration of the final hypothesis. We thus computed the RR composite as a product of the degree of increases in speech and social interaction, along with decreases in simple symbolic play, by using the following equation:

$$RR=\Delta {Sp}_{2}/\Delta t-(\Delta S_{ymb2}/\Delta t-\Delta S_{ymb3}/\Delta t)$$

**Fig 7:**
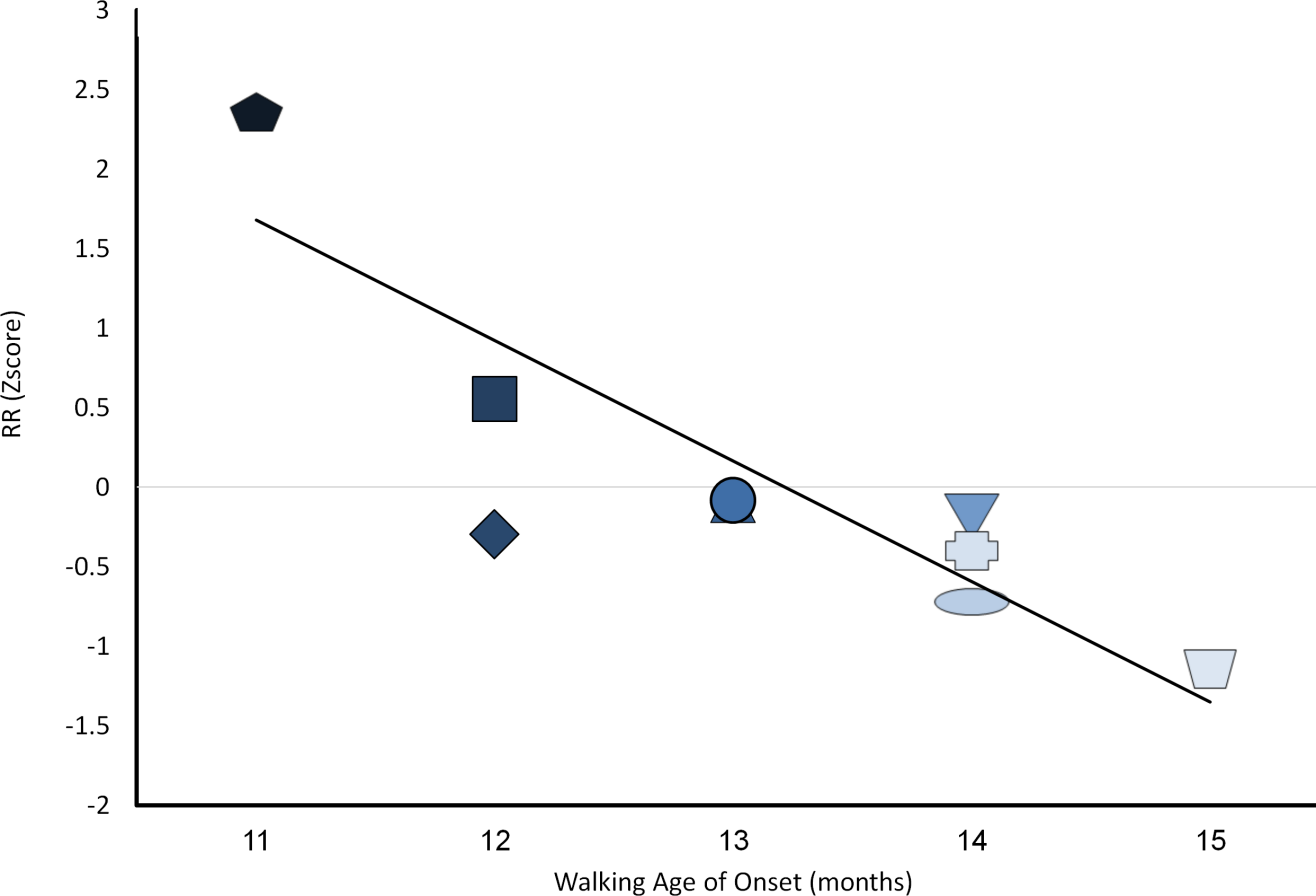
Relations between changes in cognitive resource allocation and the age of walking. Darker color shade for participants (N = 9) represents earlier age of walking initiation. Y-axis depicts standard Z scores.

Correlations between *RR* and age of walking initiation yielded an even stronger relationship (r = .906***, p < .001; Fig 7), with now only 1 participant's scores shying a bit away from the linear trajectory, thereby providing support for the final hypothesis. The relationship between the degree of resource reallocation and age of initiation of walking was such that greater RR was related to earlier walking initiation. This was evident using the current algorithm which measured the difference beween the degree of change in speech use at the time of walking (as compared with a previous level of speech use) and earlier resource reductions in simple symbolic behavior prior to the emergenece of complex behaviors. the product of the algorithm represents the degree of available resources that may be reallocated to afford walking initiation.

## Discussion

Current findings imply that the development of novel milestones are facilitated by a self adaptable composite. This composite manages internal resource reallocation in anticipation of developmental challenges. In the current project we explored a test case for this notion. Specifically, the facilitating effects of developmental changes in representational abilities, i.e. symbolic object use and socio-communication, on walking initiation. Findings suggest that walking initiation is linked to the probability of having attained previous symbolic communicative skills, along with a *proactive reallocation* of these resources [22, 39]. This reallocation possibly serves as a mechanism for preventing over-taxing cognitive resources, thereby affording resource recruitment in order to enable earlier transition [40] to walking [22, 33].

Current findings with regard to resource reallocation may underscore two important general notions about development: first, that developments in other, seemingly unrelated skills, may set the groundwork for the development of another domain; and second, that more is not necessarily better, but rather adaptability and regulation are key for the emergence of a new milestone, such as walking. That is, the ability to modify and change frequency of use of certain behaviors may possibly preemptively prevent over-taxing, and thereby increase the probability of attaining a new developmental milestone.

More specifically, the resource reallocation (RR) notion tested in the current project suggests that expressions of capacities are possibly modified proactively. That is, some increase (or decrease less), while others decrease more in order to free resources for an upcoming new skill, very much like a zero-sum game system.

Current preliminary data seem to suggest that taking a computational approach, such as the proposed equation, may provide a quantifiable measure to explore the hypothesized notions with regard to resource allocation and resource reallocation. Indeed, current results seem to provide preliminary quantifiable support for the RR hypothesis, such that integrating changes in resource use of preceding milestones in other domains sets the groundwork for upcoming novel capacities. Pending further validation, current data may suggest that RR predicts the emergence of walking better than unitary change indexes that were explored.

This notion may make sense particularly in the context of development, in which multiple processes co-occur. As such, we suggest that rather than trying to look at each capacity separately and manipulate it in order to understand its developmental trajectory; an integrative computational approach may offer an added value in certain contexts of developmental research.

These results might suggest a new productive approach for future studies with larger and more heterogeneous samples, as findings resonate with notions regarding other human populations, as well as with zoological literature and research with populations who struggle with walking. Reports have noted relations between cognitive skills (attention and dual-tasking) and walking in the elderly (for a review [41]). Current findings now indicate that these relations between cognitive and motor skills may pertain to young infants as well. Further, they provide evidence for the notion that proactive cognitive resource reallocation may serve as a potential facilitating mechanism, as current results show that such internal adaptations may occur in human infants weeks or months prior to walking’s first emergence.

These data also point to the potential usefulness of recording *changes* in frequency of use of certain behaviors, rather than noting absolute levels, as the RR notion implies that more is not necessarily better, but rather that adjustment may serve as a driving force in and of itself.

Some evolutionary literature underscore the need to reallocate resources in the evolution of bipedalism. These literature focused on external resources. This may suggest that internal resource reallocation may apply to other species, however, references to other species may also warrant some qualification of the above notions. Research with Capuchin monkeys seem to point to external resource reallocation [15], yet the report did not take into account the subject's developmental competence. Work with Chimpanzees has noted external conditions, such as presence/or absence of environmental barriers, as playing an important role in participants' symbolic behavior (e.g., use of gestures). This was thought to suggest that the presence of barriers enforces an increased use of gestures [42]. Current data may suggest that the external limiting barriers in the environment were possibly handled through the evolutionary ladder by using *internal resource reallocation*, in order to support the emergence of the novel symbolic milestone in the young Chimpanzees. This explanation may also account for the decrease in gesturing in the young chimpanzees in the Leavens et al [42] report in a manner that is compatible with the results of the current study in human infants tested in their homes.

The current study examines interactions between developmental trajectories of communication, symbolic development and motor development, all evolving in concert. Given the correlational nature of the current dataset, drawing conclusions with regard to cause-and-effect is beyond our scope. However, looking at changes before and after individual age of new milestone behaviors, current data extends developmental literature by underscoring the bi-directional nature of these inter-domain relationships. Namely, by suggesting that not only is walking related to socio-cognitive development, changes in socio-cognitive development are possibly linked to walking emergence.

More specifically, with regard to cognitive resources, current results suggest that social communication and changes in expressions of symbolic play with single and multiple objects are related to the development of walking. Thus, we are presenting a notion that symbolic object use in social contexts (symbolic play and social interaction) may be involved in the development of walking in human infants. To test this notion we explored three facets of social-communication: symbolic play development, verbal development, and dyadic interaction. Two clusters of strong relationships with onset age of walking were found; one involving symbolic play development and the other concerning symbolic language and social interaction with the mother. Findings seem to underscore all three facets of social-symbolic communication in the development of walking.

Yet, a note of qualification is warranted in this regard. The current sample was drawn from an urban, fairly western society [43]. Further research with other socio-cultural characteristics may explore the degree of universality of the current notions with regard to infants’ walking [44, 45]. Research points to socio-cultural diversity in cognitive trajectories [46] as well as in age of walking [45]. In their review, Karasik and colleagues [30] note that in several communities in Mali, and with Bambara infants, for whom caregivers massage and exercise babies as part of their daily routines, infants walk earlier. Interestingly, in these cultures a series of interactive social motor activities with the infant are employed by the infant's mother or grandmother, such as tossing the child in the air, exercising the infant [47, 48], and/or massaging the baby while bathing [47]. This data may point again to the involvement of social input relations in walking, yet offer different characteristics for the interactions. This suggests a socially mediated stimulatory sensory-motor characteristic, possibly activating somewhat different neural networks than the one conjured in the current WEIRD-like cohort (i.e., a fairly Western, Educated, Industrialized, Rich, and Democratic sample) [43]. The latter, has been suggested to present the infant with multiple restrictions and barriers [42] along with real-time parental verbal mediation; thereby possibly underscoring the usefulness of representational resources (as compared with sensory-motor ones, for example).

The current report may offer a starting point for resource reallocation research, yet further research is called for bearing in mind the limitation of these data. First, current findings rely on a modest sample size of 9 participants. Replication with larger more heterogeneous samples may yield insights concerning the generalizability of these ideas. Second, the current, fairly extensive follow-up, lasted a year accompanying the infants up to 18 months of age. Longer follow-up protocols are recommended that continue well after walking becomes automatic and requires less resources. Given the expected rebound seen in Fig 2 –underscoring the notion that the next developmental challenge may require a similar RR mechanism- this notion is testable with extended follow-up. Third, current data seem to support the usefulness of representational resources in voluntary walking initiation [1, 7, 49]. Further research may explore the necessary and sufficient resources to enable involuntary walking as well. Fourth, the current sample was quite homogenous in terms of familial resources, as well as the children's capacities. All families came from typical mid-socio-economic backgrounds in an urban environment. Further, all children performed within normal limits for their ages and backgrounds, initiating symbolic play levels, communication and walking within one SD of their expected mean. Still, exploration of individual differences, presented in Figs 3–6, underscore significant variation even within such a modest sample. Further work drawn from the tails of the current distribution may enable exploration of the limits of the relationships seen.

Previous work with children who struggle with socio-communicative issues, for example, seem to show delayed emergence of walking. Children with autism spectrum disorder were shown to initiate walking later than controls [50]. Also children with genetically-based verbal communication disability, but with otherwise preserved skills seem to initiate walking later than controls [51]. A similar trend was noted in environmental socio-communicative deprivation, namely in infants who were raised in environments with low socio-communicative enrichment, such as in cases of institutionalization [52]. Integrating the genetic and environmental sources of social-communication deficits and later emergence of walking could support current findings, yet both genetic and environmental models lack specificity, as a pervasive developmental influence cannot be ruled-out. Further work exploring in greater depth the trajectories of symbolic abilities in at-risk samples prospectively may help deepen our understanding of the proposed RR notion in other cohorts and test its limits.

Finally, exploring diverse socio-cultural cohorts, integrating neuroscience, evolutionary research and intelligent robotics [53] may further support the notions presented in the current study. These works may explore models that permutate environmental resources, internal representational skills, attention, a problem-solving apparatus, as well as sensory-motor social inputs, in order to explore reciprocal and causal relationships. Understanding these relations may yield higher probabilities for the emergence of walking and kindle improved preventative means of sub-optimal walking patterns in challenging conditions.
